# *Legionella micdadei*: A Forgotten Etiology of Growing Cavitary Nodules: A Case Report and Literature Review

**DOI:** 10.1155/2015/535012

**Published:** 2015-09-15

**Authors:** Daniel Lachant, Paritosh Prasad

**Affiliations:** ^1^Division of Pulmonary/Critical Care Medicine, Strong Memorial Hospital, University of Rochester Medical Center, 601 Elmwood Avenue, P.O. Box 692, Rochester, NY 14620, USA; ^2^Division of Transplant Infectious Disease/Critical Care Medicine, Strong Memorial Hospital, University of Rochester Medical Center, 601 Elmwood Avenue, P.O. Box 692, Rochester, NY 14620, USA

## Abstract

*Background*. *Legionella micdadei* is a Gram negative bacterium that can stain weakly acid fast. It was first described in 1979 after immunosuppressed patients developed pneumonia at a Pittsburgh VA, initially given the name Pittsburgh Pneumonia Agent. It is the second most common *Legionella* species causing infection after *pneumophila*, and typically infects immunocompromised hosts. It is not easy to be cultured which makes diagnosing difficult. *Case Presentation*. A 31-year-old female with ulcerative colitis, primary sclerosing cholangitis, and cirrhosis presented with fever, chills, shortness of breath, dry cough, and chest pain for five days after being started on immunosuppression for autoimmune hepatitis two months earlier. The first chest CT showed small bilateral cavitary nodules. The nodules continued to grow on subsequent imaging despite what was thought to be appropriate therapy. A transthoracic biopsy was performed which grew *Legionella micdadei* and the patient improved after being treated with levofloxacin. *Conclusion*. *Legionella micdadei* is an atypical pathogen known to cause pneumonia in immunosuppressed patients. This case highlights a typical presentation of an atypical infection not commonly thought about and should be considered when nodules are growing despite being on broad antimicrobial therapy.

## 1. Background

*Legionella micdadei* is a Gram negative bacterium [1]. It was first described in 1979 as a weakly acid-fast bacterium found in immunocompromised patients at a Pittsburgh VA, later identified as Pittsburgh Pneumonia Agent [1–3]. Presentation varies and includes fever, respiratory symptoms, pleuritic chest pain, lethargy, and altered mental status typically infecting immunocompromised patients [1, 4]. It is the second most common* Legionella* species, after* pneumophila*, comprising about 9% of cases [1]. Radiographic appearance in immunocompromised patients can have nodular infiltrates with a tendency to cavitate and enlarge [1, 2, 4]. We present this case to highlight and reinforce that* L. micdadei* should be on the differential in the workup of pulmonary nodule in immunocompromised patients, and with its difficulty being cultured from respiratory secretions, lung tissue may help with diagnosis.

## 2. Case Presentation

**Figure 1:**
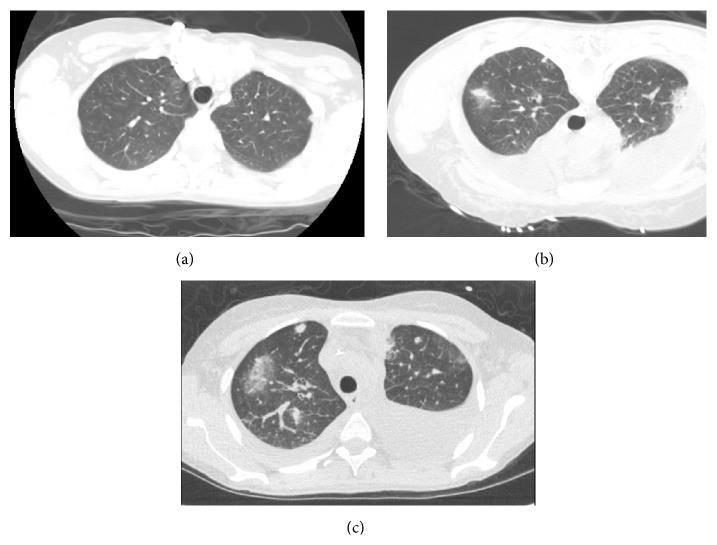
The CT images are taken roughly from the same level. (a) The CT scan on admission. (b) The CT image from the CT guided biopsy showing new nodules. Note that the patient is prone in this image. (c) The CT image is 9 days after admission and shows continued growth and new nodules.

A 31-year-old Caucasian female with ulcerative colitis, primary sclerosing cholangitis, and cirrhosis presented with fever, chills, shortness of breath, dry cough, and chest pain for five days after being started on prednisone 40 mg daily and azathioprine 50 mg daily for autoimmune hepatitis two months earlier. She was born and raised in New York state and only travelled to Vermont and Florida. During the prior two weeks, she traveled to a hunting shack in northern New York and had been there twice before becoming ill. She had no personal or family history of tuberculosis and has had several prior negative PPDs during nursing school. On presentation, she was febrile to 102°F, pulse of 120 beats per minute, respiratory rate of 20 breaths per minute, and blood pressure of 96/58 mmHg and required 2 liters oxygen to keep her saturation above 90%. Initial exam was benign with clear lungs. After blood and urine cultures were obtained, vancomycin and meropenem were started empirically. A paracentesis was not performed due to small ascitic fluid pockets. Initial chest X-ray showed trace left-sided pleural effusion without any evidence of infiltrates. Initial lab work showed ALT 62 U/L, AST 87 U/L, alkaline phosphatase 158 U/L, total bilirubin 13.2 mg/dL, INR of 2.9, WBC of 16,000/ul with 14,400/uL neutrophils, hemoglobin 7.8 g/dL, and platelets of 167,000/uL. After a CT chest revealed small bilateral cavitary nodules the following day, vancomycin and meropenem were continued along with amphotericin being started with concern for a fungal infection (Figure 1).

Without clinical improvement, a CT guided biopsy of the necrotic pulmonary nodules was performed by interventional radiology three days after admission. The CT chest of the biopsy showed a new right upper lobe lesion (Figure 1) despite being on meropenem, vancomycin, and amphotericin. A bronchoscopy with bronchoalveolar lavage was performed the following day and Gram stain, fungal stain, and extensive cultures were all negative. With continued deterioration six days after the biopsy, a repeat CT chest showed increasing size and number of pulmonary nodules with ground glass opacities despite treatment with meropenem, vancomycin, and amphotericin. On day eleven of admission, cultures from the CT guided biopsy turned positive for* Legionella*. The antimicrobials were stopped and levofloxacin was started and continued for 3 weeks with resolution of symptoms. The culture was sent to the New York State Department of Health in Albany, NY, and identified as* L. micdadei*. Negative tests included normal transthoracic echo, normal head CT, and normal neck ultrasound. Five blood cultures, respiratory viral panel, urine* Legionella* antigen,* Pneumocystis* DNA PCR,* Legionella* culture from BAL, bacterial culture, fungal culture,* Aspergillus* antigen, and* Histoplasma* antigen were negative.

## 3. Discussion

This case reinforces and highlights the importance of recognizing a typical pattern of an atypical disease as* L. micdadei* was first described as pulmonary nodules in immunocompromised patients. Pulmonary nodules are common in immunocompromised patients and account for significant morbidity and mortality [5]. The nodule etiology varies by degree of immunosuppression, prophylaxis, exposures, and medical history [6] making them difficult for diagnosis [3]. The infectious differential includes invasive fungal diseases which are the most common cause (e.g.,* Aspergillus*,* Cryptococcus*, and Zygomycetes),* Nocardia*,* Legionella*, mycobacterial infection, viral (e.g., CMV, adenovirus), septic emboli, or endocarditis. [6]. Once infiltrates are identified on radiograph fiberoptic bronchoscopy with lavage and possible transbronchial biopsy should be undertaken early and when safe to improve identification of the causative organism to help ensure proper and narrow antimicrobial coverage [5].

*Legionella micdadei* is a fastidious aerobic Gram negative bacterium naturally found in water and soil [4]. Despite being a Gram negative, it can stain weakly acid fast and initially can be confused for* Mycobacterium* [2, 7]. With its poor counterstain uptake, Gram stains typically show polymorphonuclear cells without bacteria [4, 7]. In our patient, the Gram stain from the CT guided biopsy confirmed this and only contained 10–25 polymorphonuclear cells/low power field without organism while her bronchoalveolar lavage had no polymorphonuclear cells or organisms seen. The discrepancy with our lavage and biopsy could be the difficulty in isolating* Legionella* or more likely despite being in the correct airway the lavage fluid was not near a site of infection. It is the second most common* Legionella* species behind* pneumophila* with an incidence of about 9% of all* Legionella* cases, although its true prevalence is unknown due to its difficulty in culturing [4]. The* Legionella pneumophila* urine antigen does not turn positive with* L. micdadei* infections and cannot rule out the infection [4, 7], as seen with our patient.

It was first reported as an invasive pulmonary pathogen in 1979 called Pittsburgh Pneumonia Agent after infecting a total of 8 patients in Pittsburgh and 5 patients in Virginia [2, 3, 7]. At the time, it was considered an opportunistic infection after six renal transplant patients, one chronic lymphocytic leukemia patient, and one cutaneous herpes zoster patient in Pittsburgh had received high dose steroids [2, 3]. Since then, this infection has been identified in other immunocompromised populations including HIV and bone marrow transplantation [7]. The organism was isolated from lung tissue in all 8 cases and was noted to be difficult to stain and culture [2], as it grows on buffered charcoal yeast extract and not typical media with it lacking beta-lactamase [4].

*L. micdadei* infections occur more commonly in immunocompromised patients [4]. Presentations vary and typically include fever, altered mental status, lethargy, pleuritic chest pain, respiratory, and gastrointestinal symptoms [1, 4]. In the original description of* L. micdadei,* the radiographic appearance consisted of patchy alveolar infiltrates in four patients and nodular densities ranging in size from 2 to 4 cm in another four patients with progression on imaging despite broad antibiotics [2]. At the time, cavitations were not visualized radiographically but were seen on autopsy in 2 patients [2]. Since then, immunocompromised patients have been found with* L. micdadei* causing round or nodular infiltrates that can cavitate [4]. Our patient shows a similar pattern of progression of pulmonary nodules despite being on broad antimicrobial coverage (vancomycin, meropenem, and amphotericin) all of which are ineffective against* L. micdadei* and should have prompted more thought as to what organism was not being covered. Today, the mainstay of treatment is fluoroquinolones [4], and as soon as she was started on proper therapy her symptoms improved.

This case highlights the importance of recognizing a rare etiology in a typical presentation as it was originally described. With the increasing number of immunosuppressed patients found to have pulmonary nodules, it is important for the etiology to be elucidated so proper treatment can be initiated to improve outcomes. The original description of* L. micdadei* causing enlarging pulmonary nodules with the ability to cavitate despite being on broad antimicrobial coverage is identical to our case. This organism is difficult to isolate as bronchoscopy with bronchoalveolar lavage was performed in our patient at the same time as the biopsy and did not isolate the organism. A high index of suspicion for* L. micdadei* should be held for pulmonary nodules or rapidly growing nodules despite being on broad antimicrobial coverage in an immunosuppressed patient.

## References

[1] Medarov B. I., Siddiqui A. K., Mughal T., Moshiyakhov M., Rossoff L. J. (2004). *Legionella micdadei* infection presenting as severe secretory diarrhea and a solitary pulmonary mass. *Clinical Infectious Diseases*.

[2] Myerowitz R. L., Pasculle A. W., Dowling J. N. (1979). Opportunistic lung infection due to ‘Pittsburgh pneumonia agent’. *The New England Journal of Medicine*.

[3] Rogers B., Donowitz G., Walker G., Harding S., Sande M. (1979). Opportunistic pneumonia: a clinicopathological study of five cases caused by an unidentified acid-fast bacterium. *The New England Journal of Medicine*.

[4] Muder R. R., Yu V. L. (2002). Infection due to Legionella species other than *L. pneumophila*. *Clinical Infectious Diseases*.

[5] Jain P., Sandur S., Meli Y., Arroliga A. C., Stoller J. K., Mehta A. C. (2004). Role of flexible bronchoscopy in immunocompromised patients with lung infiltrates. *Chest*.

[6] Waldron P. R., Martin B. A., Ho D. Y. (2015). Mistaken identity: *Legionella micdadei* appearing as acid-fast bacilli on lung biopsy of a hematopoietic stem cell transplant patient. *Transplant Infectious Disease*.

[7] Kaul D. R., Riddell J. (2009). Approach to the immunocompromised patient with pulmonary nodules. *Current Fungal Infection Reports*.

